# Identification of hub genes for the diagnosis and prognosis in triple negative breast cancer using transcriptome and differential methylation integration analysis

**DOI:** 10.7150/jca.104472

**Published:** 2025-03-03

**Authors:** Baoe Liu, Xiaoli Yang, Huxia Wang, Peijun Liu, Qing Feng, Cuixiang Xu, Zhangjun Song

**Affiliations:** 1 Department of Breast Disease Center, Shaanxi Provincial People's Hospital, Xi'an 710068, Shaanxi, China.; 2 Shaanxi Provincial Key Laboratory of Infection and Immune Diseases, Shaanxi Provincial People's Hospital, Xi'an, Shaanxi, China.; 3 Department of Breast Disease Center, Shaanxi Provincial Tumor Hospital, Xi'an, Shaanxi, China.; 4 The first affiliated hospital, Health Science Center, Xi'an Jiaotong University, Xi'an, Shaanxi, China.; 5 Department of Oncology, Shaanxi Provincial People's Hospital, Xi'an 710068, Shaanxi, China.

**Keywords:** triple negative breast cancer, transcriptome, DNA methylation, diagnosis, prognosis

## Abstract

**Introduction:** Triple-negative breast cancer (TNBC) is characterized by the absence of estrogen receptor (ER), progesterone receptor (PR), and human epidermal growth factor receptor 2 (HER2) expression. It is highly invasive and aggressive, making it the subtype of breast cancer with the poorest prognosis. Currently, systemic chemotherapy is the primary treatment option, but targeted therapies remain unavailable. Therefore, there is an urgent need to identify novel biomarkers for the early diagnosis and treatment of TNBC.

**Methods:** We conducted an integrated analysis of transcriptome and methylation data to identify methylation-regulated differentially expressed genes (MDEGs). Gene Ontology (GO) analysis, Kyoto Encyclopedia of Genes and Genomes (KEGG) pathway analysis, and protein-protein interaction (PPI) network analysis were performed on MDEGs to investigate the impact of hub genes on the diagnosis and prognosis of TNBC. Subsequently, the expression levels and DNA methylation patterns of key genes were validated in the TNBC cell line MDA-MB-231 and the normal breast epithelial cell line MCF-10A using reverse transcription quantitative PCR (RT-qPCR) and quantitative methylation-specific PCR (qMSP).

**Results:** A total of 98 upregulated and 87 downregulated genes were identified through transcriptomic profiling integration analysis. By incorporating methylation data, we further identified 22 genes with high expression of hypomethylation (hypo-MDEGs) and 32 genes with low expression of hypermethylation (hyper-MDEGs). The hypo-MDEGs were primarily involved in nuclear division, organelle fission, spindle formation, chromosome and kinetochore development, and protein binding. KEGG pathway analysis revealed that these genes were enriched in progesterone-mediated oocyte maturation, cell cycle regulation, and oocyte meiosis. Hyper-MDEGs were associated with cell proliferation, hormone response, pain, extracellular matrix composition, and binding to sulfur compounds, heparin, and glycosaminoglycans. PPI network analysis identified seven hub genes—*EXO1, KIF11*, *FOXM1, CENPF, CCNB1*, *PLK1*, and *KIF23*—which were all significantly overexpressed in TNBC tissues and positively correlated with each other (*p* < 0.05). Receiver operating characteristic curve analysis showed that the area under the curve (AUC) for all seven genes exceeded 0.9 (*p* < 0.05), suggesting strong diagnostic potential. Kaplan-Meier survival analysis indicated that *KIF11*, *CCNB1*, and *PLK1* were associated with a higher hazard ratio (HR > 1, *p* < 0.05) in TNBC. *In vitro* validation experiments demonstrated that, compared to MCF-10A cells, MDA-MB-231 cells exhibited higher mRNA expression levels of *KIF11*, *CCNB1*, and *PLK1*, while their DNA methylation levels were lower. Conclusions: This study identified seven hypo-MDEGs, including *EXO1, KIF11*, *FOXM1, CENPF, CCNB1*, *PLK1*, and *KIF23,* which are involved in the regulation of the cell cycle and mitotic processes and have significant potential as diagnostic biomarkers for TNBC. Notably, elevated expression of *KIF11*, *CCNB1*, and *PLK1* is associated with poor prognosis in patients with TNBC. These findings contribute to an improved understanding of the epigenetic molecular mechanisms underlying TNBC progression and highlight novel biomarkers that may enhance the accuracy of TNBC diagnosis and provide potential targets for therapeutic intervention.

## 1. Introduction

Breast cancer is the most prevalent malignancy and the second leading cause of cancer-related mortality among women worldwide, accounting for approximately 13% of all cancer-related deaths annually, according to the World Health Organization 1. It is a highly heterogeneous tumor that is clinically classified into four distinct subtypes 2, based on the expression patterns of immunohistochemical markers, including estrogen receptor (ER), progesterone receptor (PR), human epidermal growth factor receptor 2 (HER2), and KI-67. Triple-negative breast cancer (TNBC) is characterized by the absence of ER, PR, and HER2 expression and is considered the most aggressive and malignant subtype of breast cancer 3, accounting for approximately 15%-25% of all invasive breast cancers 4. Due to the lack of targeted therapies 5, systemic chemotherapy remains the primary treatment option; however, its effectiveness is limited by high systemic toxicity and multidrug resistance 6. Consequently, there is an urgent need to identify novel biomarkers for the early diagnosis and targeted treatment of TNBC.

Epigenetics refers to heritable changes in gene expression that do not involve alterations in the DNA sequence but rather a regulatory code that governs gene function 7. Among various epigenetic modifications, DNA methylation is the most extensively studied and plays a crucial role in genomic regulation, occurring primarily at the 5' site of CpG dinucleotides 8. An increasing number of studies have demonstrated that aberrant DNA methylation is a key contributor to breast cancer onset, metastasis, and prognosis 9. For instance, a study found that elevated levels of gene promoter methylation were strongly associated with hormone receptor-positive breast tumors, and hypermethylation of *FZD9*, *MME*, *BCAP31, HDAC9*, *PAX6*, *SCGB3A1*, and *PDGFRA* genes effectively predicted hormone receptor-positive breast cancer 10. Furthermore, SO JY *et al.* reported that DNA methyltransferase 3B mediates the epigenetic programming of metastatic breast cancer cells by altering multiple signaling pathways, including STAT3, NF-κB, PI3K/Akt, β-catenin, and Notch signaling 11. Given that DNA methylation is a reversible process, it presents a promising therapeutic target for the intervention and treatment of breast cancer.

Evidence suggests that abnormal DNA methylation plays a critical role in the development of TNBC. For example, a study conducted at Lund University in Sweden 12 reported an elevated incidence of *BRCA1* hypermethylation in early-stage TNBC, indicating an association between *BRCA1* hypermethylation and the onset of early-stage TNBC 13. Another study demonstrated that promoter hypomethylation of three breast cancer stem cell-related genes—*CD44*, *CD133*, and *MSH1*—was strongly correlated with TNBC aggressiveness 14. In addition, aberrant DNA methylation of specific genes has been linked to both the initiation and progression of TNBC 15. DNA methylation has also been identified as a potential prognostic biomarker for TNBC. Mendaza *et al.* 16 suggested that ADAM12 hypomethylation was associated with poor prognosis in patients with TNBC. A bioinformatics analysis 17 integrating mRNA expression and promoter methylation demonstrated that PLA2R1 downregulation, accompanied by promoter hypermethylation, was observed in TNBC. This finding suggests that PLA2R1 may serve as a valuable diagnostic and prognostic biomarker. Additionally, another study reported a significant link between hypomethylation and abnormal activation of the *CT83* gene in TNBC 18, which was associated with a reduced overall survival rate in patients with breast cancer. Previous research on gene methylation in TNBC has been limited, with most studies focusing on the effects of methylation on individual genes or small gene subsets. As a result, there remains a substantial knowledge gap regarding the genome-wide impact of DNA methylation on TNBC development and progression. Therefore, it is essential to investigate the role of aberrant DNA methylation in TNBC using a comprehensive genomic approach.

**Figure 1:**
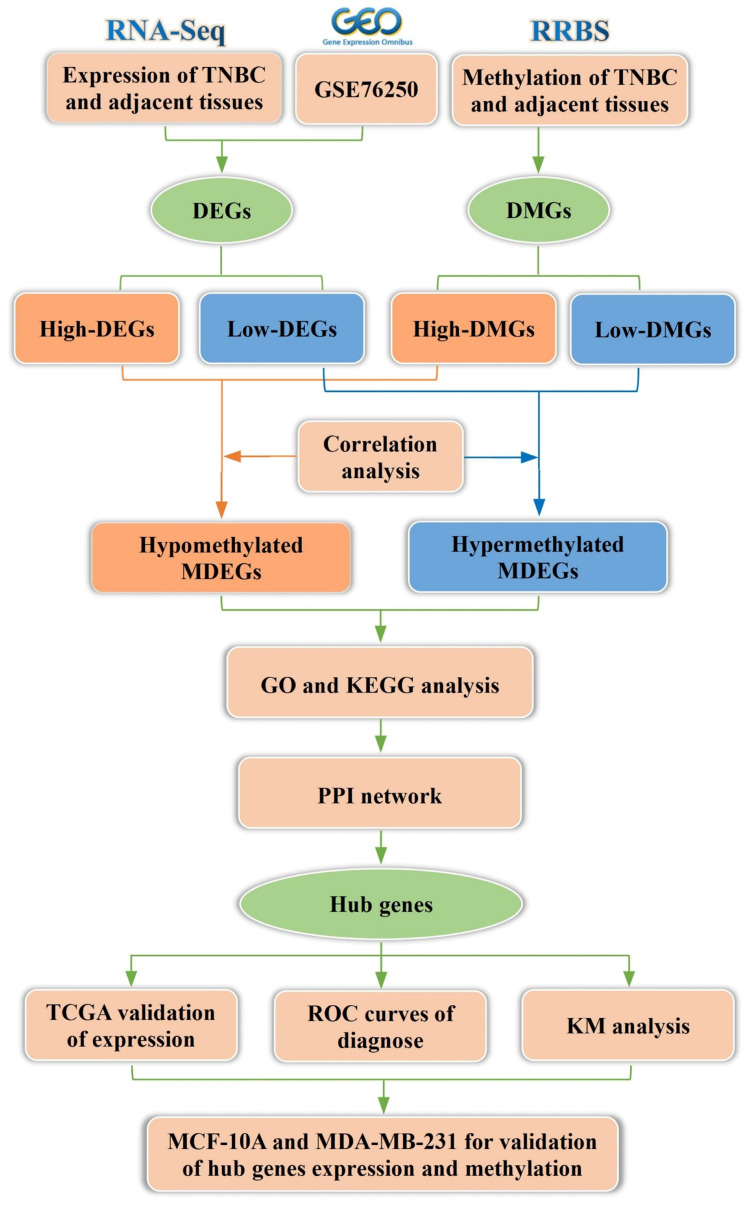
Flowchart of bioinformatics analysis. TNBC: triple-negative breast cancer, DEGs: differentially expressed genes, DMGs: differentially methylated genes, MDEGs: methylation-regulated differentially expressed genes. GSE76250: the transcriptome profiling data in TNBC.

In the present study, we conducted an integrated analysis of transcriptomic and methylation data to identify methylation-regulated differentially expressed genes (MDEGs). Gene Ontology (GO) analysis, Kyoto Encyclopedia of Genes and Genomes (KEGG) pathway analysis, and protein-protein interaction (PPI) network analysis were subsequently performed on MDEGs to identify key genes. The expression levels of these key genes were further analyzed using data from The Cancer Genome Atlas (TCGA, https://tcga-data.nci.nih.gov/tcga/). Additionally, we evaluated the relationship between key genes and TNBC diagnosis and prognosis. Finally, the expression of key genes was validated in the TNBC cell line MDA-MB-231 and the normal breast epithelial cell line MCF-10A. This study may provide valuable insights into the diagnosis and precision treatment of TNBC. The workflow of the analysis is illustrated in Figure 1.

## 2. Materials and Methods

### 2.1 Patients and tissue specimens

The cases in this study were obtained from primary patients with TNBC who underwent radical mastectomy at Shaanxi Provincial People's Hospital, with a total of six patients with TNBC included. Cancer tissues and paired adjacent noncancerous tissues were collected from these patients, and tissue DNA and RNA were extracted. Reduced representation bisulfite sequencing (RRBS) and whole genome RNA sequencing (RNA-seq) were performed on six cancer tissues and six adjacent noncancerous tissue samples. This study was approved by the Medical Ethics Committee of Shaanxi Provincial People's Hospital (2023-R168), and all participants provided written informed consent. Additionally, the external dataset GSE76250, a transcriptomic sequencing dataset, was used for validation and contains 165 TNBC samples and 33 adjacent noncancerous tissues.

### 2.2 Acquisition and processing of expression data

Total RNA was isolated and purified from tissue samples using TRIzol™ reagent (Invitrogen, CA, USA). PolyA mRNA was selectively captured using Dynabeads™ Oligo (dT) (Thermo Fisher, CA, USA). The captured mRNA was subsequently fragmented using the Magnesium RNA Fragmentation Module (New England Biolabs, CA, USA) and then reverse-transcribed to synthesize complementary DNA (cDNA) using SuperScript™ II Reverse Transcriptase (Invitrogen, CA, USA). Following PCR amplification, the average insert length of the cDNA library was 300 ± 50 bp. Paired-end (2 × 150 bp) sequencing was performed on an Illumina NovaSeq™ 6000 (LC-Bio Technology Co, Hangzhou, China) following the manufacturer's recommended protocol. Fastp software was used to filter the raw sequencing reads by removing those containing adapter contamination, low-quality bases, or undetermined bases using default parameters. Differentially expressed genes (DEGs) were identified using parametric F-tests comparing nested linear models in the R package edgeR. DEGs were selected based on the criteria of |log₂ fold change (log₂FC)| ≥ 1 and adjusted *p*-value < 0.05.

The expression matrix of GSE76250 and its corresponding platform information were downloaded and processed from the Gene Expression Omnibus (GEO, https://www.ncbi.nlm.nih.gov/geo/) database using the GEOquery package in R 4.2.0. We extracted gene expression profiles and clinicopathological data from 33 patients with TNBC, including paired carcinoma and adjacent normal tissues. Data normalization and differential expression analysis were performed using the limma package in R, and DEGs were identified based on the criteria of adjusted *p*-value < 0.05 and |log_2_FC| ≥ 1. The intersection of differentially expressed gene lists from multiple datasets was used for subsequent analyses.

### 2.3 Methylation detection and processing of the results

Total DNA was extracted from tissue samples using the QIAamp Rapid DNA Tissue Kit (Qiagen, Düsseldorf, Germany) following the manufacturer's protocol. DNA fragmentation was performed using the MspI restriction enzyme (New England Biolabs, CA, USA), followed by bisulfite conversion. Fragmented DNA (200-500 bp) was selected and subjected to PCR amplification to construct sequencing libraries. Paired-end (2 × 150 bp) sequencing was conducted on an Illumina NovaSeq™ 6000 platform. For raw sequencing reads, Cutadapt was used to remove reads containing adapter contamination, low-quality bases, and undetermined bases. Sequence quality was verified using FastQC, and reads passing quality control were aligned to the reference genome using Bismark. After alignment, duplicate reads were removed using Samtools to eliminate redundancy. DNA methylation levels were calculated as the ratio of methylated cytosine (C) reads to total cytosine reads (methylated and unmethylated) using in-house Perl scripts and MethPipe. Differentially methylated regions were identified using the MethylKit package in R with default parameters (1,000 bp sliding windows, 500 bp overlap, *p* < 0.05). Differentially methylated genes (DMGs) were then identified using R 4.2.0, based on the criteria of adjusted *p*-value < 0.05 and |log₂FC| ≥ 1.

### 2.4 The conjoint analysis of methylome and transcriptome

The intersection of DEGs and DMGs was analyzed and visualized using the ggplot2 and VennDiagram packages in the R programming environment. Overlapping genes were identified as MDEGs. GraphPad Prism software was used to analyze the correlation between mRNA expression and methylation levels of the overlapping gene sets. Genes with high expression of hypomethylation (hypo-MDEGs) and genes with low expression of hypermethylation (hyper-MDEGs) were selected for subsequent studies.

### 2.5 Assessment of biological variables among MDEGs

To investigate the biological significance of MDEGs, GO and KEGG pathway enrichment analyses were performed separately for hypo-MDEGs and hyper-MDEGs using the clusterProfiler package in R. Enrichment results were visualized based on statistical significance (p-value) using the ggplot2 package in R.

### 2.6 Construction of PPI network and analysis of hub genes

To assess the functional interactions among gene sets in patients with TNBC, a PPI network was constructed using STRING 12.0 (https://cn.string-db.org/). The network data were imported into Cytoscape (version 3.7.1) for visualization and enhancement. Hub genes were identified using the CytoHubba application within Cytoscape, employing the MCC, MNC, Degree, Closeness, and Radiality algorithms. In addition, GraphPad Prism was used to evaluate the correlation between the expression levels of these genes, and the results were visualized using the igraph package in R.

### 2.7 Analysis of hub genes expression, diagnosis and prognosis

Raw gene expression data and clinical information for 126 patients with TNBC from The Cancer Genome Atlas (TCGA) were obtained using the TCGAbiolinks and SummarizedExperiment packages in RStudio. After quality control, differential expression analysis of hub genes was performed on 115 TNBC tumor samples and 11 adjacent noncancerous tissues using the DESeq2 package in R. The expression values of hub genes were extracted from TCGA data and subjected to statistical analysis and visualization using GraphPad Prism (version 9.0.0).

To assess the diagnostic potential of hub genes, receiver operating characteristic (ROC) curves were generated, and the area under the curve (AUC) was calculated using MedCalc (version 15.2.2). For prognostic analysis, the Kaplan-Meier (KM) plotter online tool was used to evaluate overall survival (in months) based on hub gene expression in patients with TNBC from the TCGA database.

### 2.8 Detection of expression and methylation levels of hub genes for MDA-MB-231

The TNBC cell line MDA-MB-231 and the normal breast epithelial cell line MCF-10A were used to validate the expression of hub genes. Both cell lines were gifted by Professor Liu Peijun from The First Affiliated Hospital of Xi'an Jiaotong University and certified by professional institutions. MDA-MB-231 cells were cultured in Leibovitz's L-15 medium supplemented with 10% fetal bovine serum and 1% penicillin-streptomycin. MCF-10A cells were cultured in DMEM/F12 medium containing 5% horse serum, 20 ng/mL epidermal growth factor, 0.5 μg/mL cortisol, 10 μg/mL insulin, and 1% penicillin-streptomycin.

Total RNA was extracted using the M5 HiPer Universal RNA Mini Kit (Mei5bio, Beijing, China) and reverse transcribed into cDNA using the M5 Super qPCR TR Kit with gDNA remover (Mei5bio, Beijing, China). Primers for the hub genes were designed online using Integrated DNA Technologies, and the primer sequences are provided in Table S1. Quantification of hub gene transcript levels was performed using a reverse transcription quantitative PCR (RT-qPCR) kit (Mei5bio, Beijing, China). The reaction mixture contained 10 µL Realtime PCR Super Mix, 7.4 µL ddH₂O, 1 µL cDNA template, and 0.8 µL of each primer. RT-qPCR amplification was carried out using a Gentier 96 thermal cycler (Tianlong, Xi'an, China) under the following conditions: 95°C for 30 s, followed by 40 cycles of 95°C for 5 s and 57°C for 30 s, with a final storage temperature of 4°C. All reactions were performed in duplicate, and the relative mRNA levels of hub genes were quantified using the 2^-∆∆Ct^ method, with *ACTB* as the reference gene.

Total DNA was extracted using a DNA Extraction Kit (TIANGEN, Beijing, China) and modified using the DNA Methylation-Gold Kit (ZYMO, CA, USA). Primers for the hub genes were designed online via the MethPrimer website, and the primer sequences are provided in Table S1. Quantitative methylation-specific PCR (qMSP) was employed to assess the methylation levels of key genes. The reaction mixture contained 10 µL Realtime PCR Super Mix, 7.4 µL ddH₂O, 1 µL modified DNA template, and 0.8 µL of each primer. Amplification was performed under the following conditions: 95°C for 30 s, followed by 45 cycles of 95°C for 5 s and 57°C for 30 s, with a final storage temperature of 4°C. The reference gene and calculation method were the same as described above.

### 2.9 Statistical analysis

R software (version 4.2.0) and GraphPad Prism (version 9.0.0) were used for data processing and statistical analysis. Spearman's rank correlation was applied to assess the correlation between DNA methylation levels and gene expression, as well as the correlation among hub gene expression levels. The Mann-Whitney U test was used to evaluate the differences in hub gene expression between TNBC samples and adjacent noncancerous tissues. The Welch's t-test was applied to compare hub gene expression levels between TNBC cells and normal breast epithelial cells. ROC curves were generated using MedCalc (version 15.2.2). The log-rank test was conducted to compare the prognostic impact of different hub gene expression levels in patients with TNBC. Statistical significance was defined as *p* < 0.05.

## 3. Results

### 3.1 Transcriptomic pattern of TNBC and adjacent noncancerous tissues

**Figure 2:**
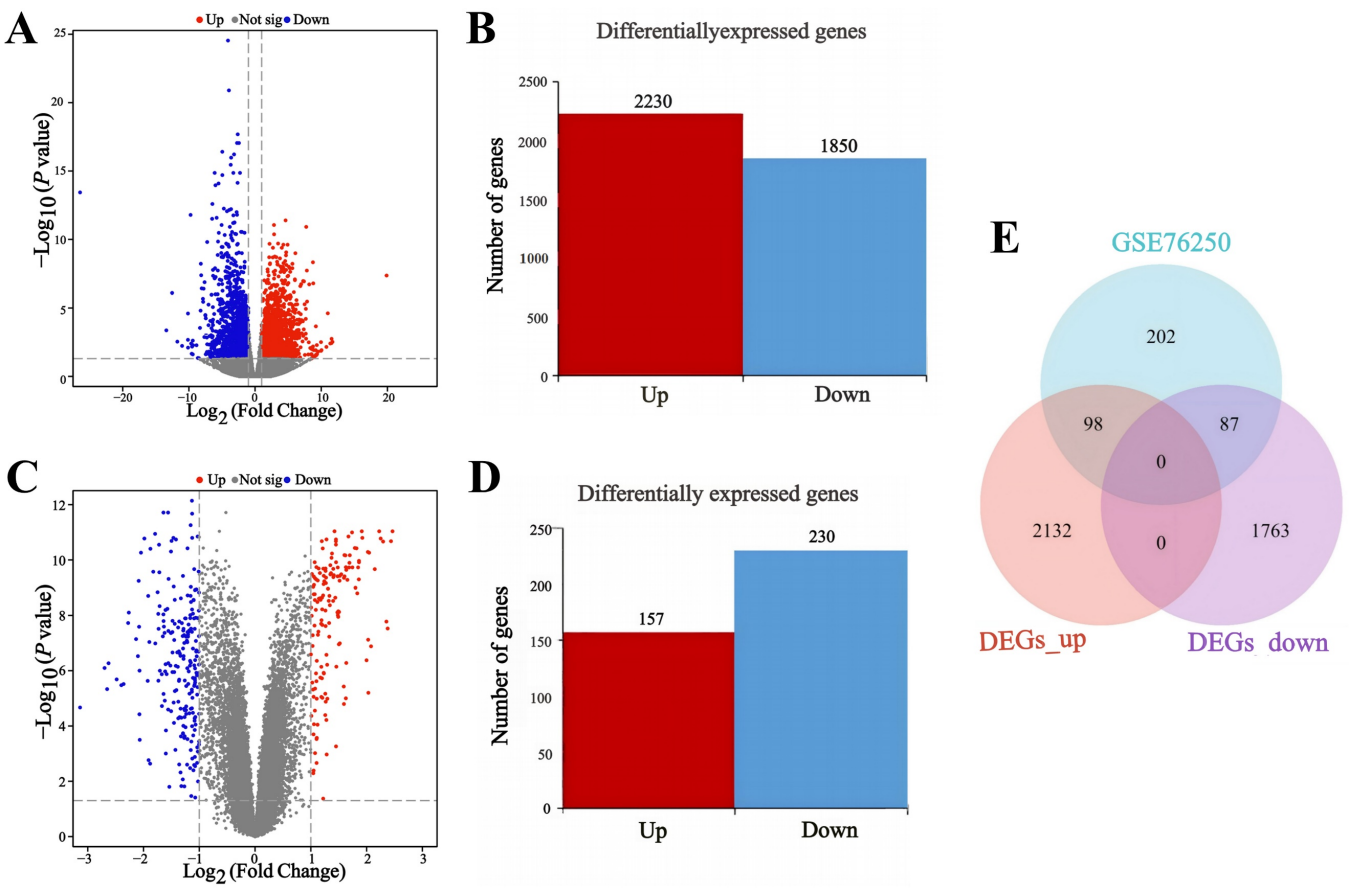
Transcriptomic pattern of TNBC and adjacent noncancerous tissues. (A) Volcano map of the gene expression levels in six pairs of TNBC and adjacent noncancerous tissues in RNA-seq. (B) Histogram of differentially expressed genes in six pairs of TNBC and adjacent noncancerous tissues. (C) Volcano plot of gene expression levels in 33 pairs of TNBC and adjacent noncancerous tissues in GSE76250 dataset. (D) Histogram of differentially expressed genes in 33 pairs of TNBC and adjacent noncancerous tissues. (E) Venn diagram of differentially expressed genes between RNA-seq group and GSE76250 dataset in TNBC. DEGs: differentially expressed genes. GSE76250: the transcriptome profiling data in TNBC.

Gene expression levels were analyzed in six pairs of TNBC and adjacent noncancerous tissues, revealing 4,080 DEGs between the two groups, as depicted in the volcano plot (Figure 2A). Among these, 2,230 genes were upregulated, and 1,850 genes were downregulated, as illustrated in the histogram (Figure 2B, Table S2). Additionally, analysis of the GSE76250 dataset identified a total of 387 DEGs between TNBC and adjacent noncancerous tissues (Figure 2C), comprising 157 upregulated genes and 230 downregulated genes (Figure 2D, Table S3). Following an integrated analysis of RNA-seq data and GSE76250 dataset, the overlapping transcriptomic data of TNBC tissues was visualized in a Venn diagram (Figure 2E), leading to the identification of 98 upregulated and 87 downregulated genes.

### 3.2 DNA methylation Levels of TNBC and adjacent noncancerous tissues

**Figure 3:**
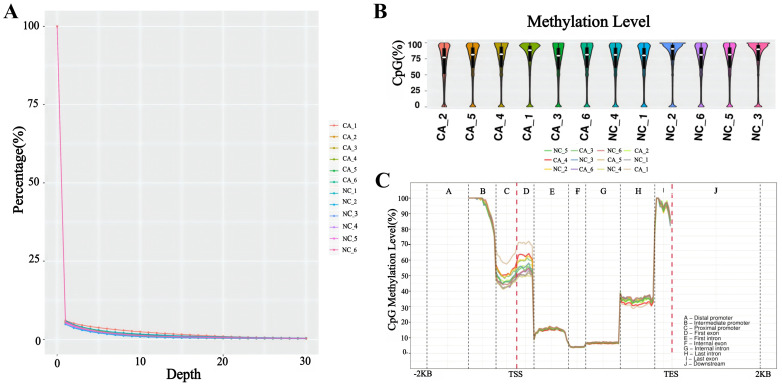
DNA methylation profiling of TNBC patients. (A) Overview of genome coverage at cytosine sites across the analyzed samples. (B) DNA methylation rates at CpG contexts across various samples. (C) Assessment of methylation levels in different gene regions across the entire gene landscape.

The DNA methylation status of various genomic regions was analyzed using RRBS. The bisulfite conversion rate exceeded 99% across all samples (Table S4). Cytosine (C) site coverage was consistent among samples (Figure 3A), confirming the completeness, accuracy, and reproducibility of the sequencing data. By comparing global methylation rates across all C sites, we found that DNA methylation predominantly occurred at CpG dinucleotides, with an average methylation level exceeding 75% (Figure 3B). Additionally, differentially methylated CpG sites were primarily located in promoter regions upstream of transcription start sites (Figure 3C).

### 3.3 Integration of methylome and transcriptomic data

**Figure 4:**
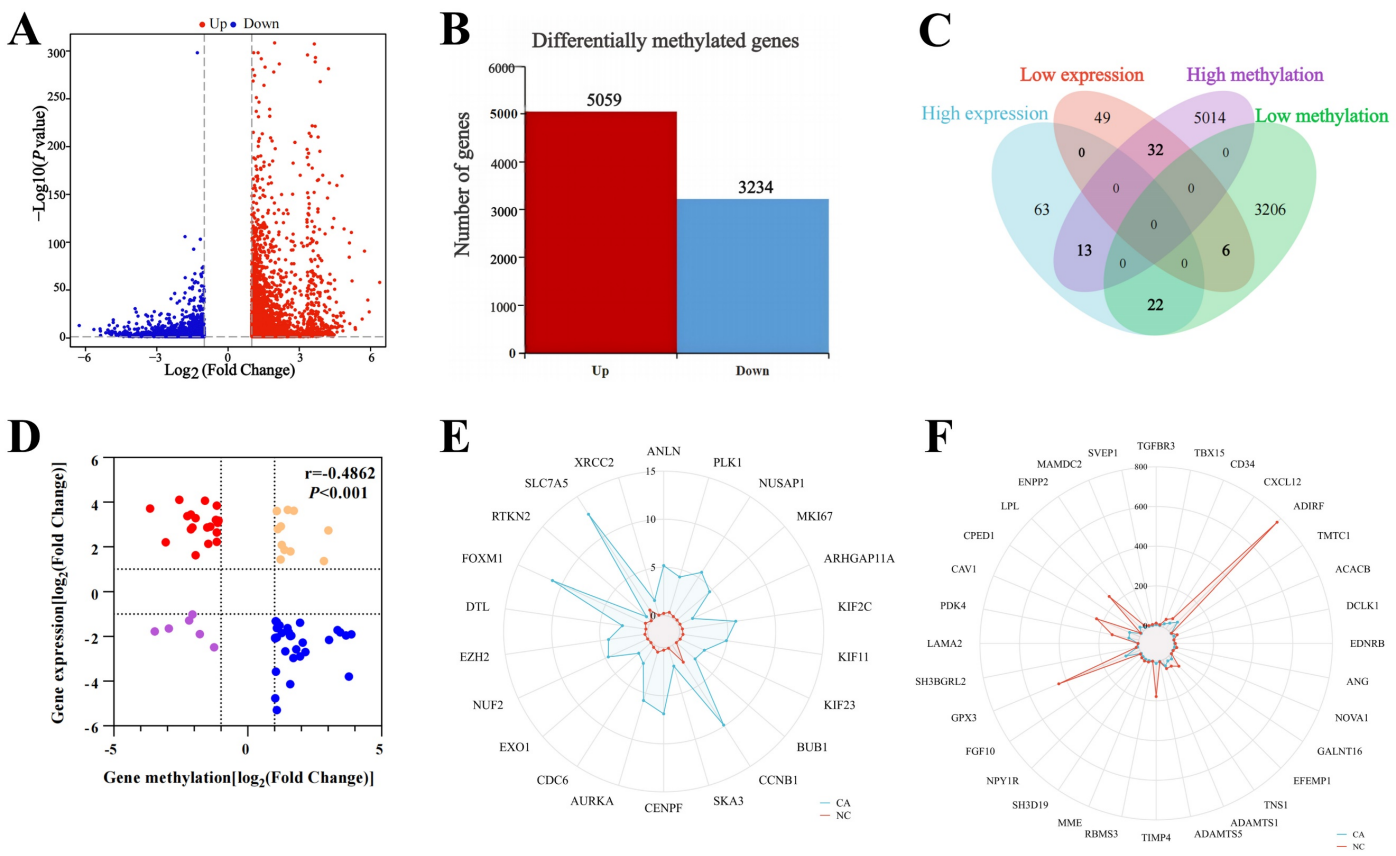
Integrative analysis of methylome and transcriptomic data in TNBC. (A) Volcano plot of genes methylation levels in six pairs of TNBC and adjacent noncancerous tissues in RRBS. (B) Histogram of DMGs in six pairs of TNBC and adjacent noncancerous tissues in RRBS. (C) Venn diagram between DMGs and DEGs of TNBC. (D) The nine-quadrant diagram of Correlation analysis between DMGs and DEGs. (E) Radar chart of 22 hypo-MDEGs. (F) Radar chart of 32 hyper-MDEGs. RRBS: reduced representation bisulfite sequencing, MDEGs: methylated differentially expressed genes.

Differential methylation analysis identified 8,293 DMGs in TNBC tissues compared with adjacent noncancerous tissues (Figure 4A). Among these, 5,059 genes were hypermethylated, while 3,234 genes were hypomethylated, as shown in the histogram (Figure 4B, Table S5). A Venn diagram illustrating the intersection of methylome and transcriptomic data from patients with TNBC identified 73 overlapping genes (Figure 4C). Correlation analysis between methylome and transcriptomic data revealed that 54 of these genes exhibited a negative correlation within the first and ninth quadrants of the nine-quadrant correlation diagram (Figure 4D). To further illustrate these findings, radar charts were used to display these 54 negatively correlated genes, including 22 hypo-MDEGs (Figure 4E) and 32 hyper-MDEGs (Figure 4F).

### 3.4 GO and KEGG enrichment analysis

**Figure 5:**
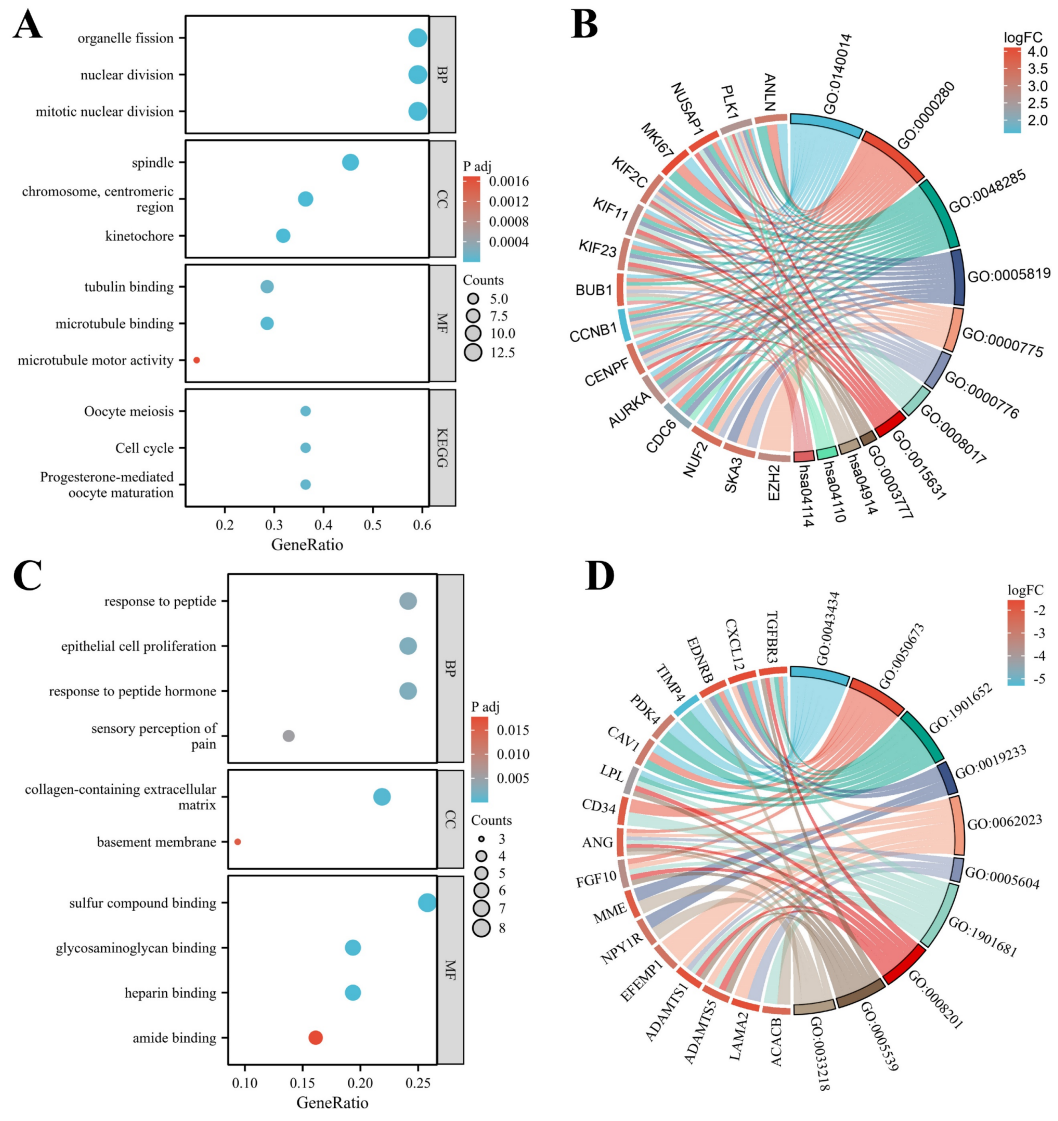
GO and KEGG enrichment analysis of hypo-MDEGs and hyper-MDEGs in TNBC. (A) Bubble chart of GO and KEGG enrichment analysis in hypo-MDEGs. (B) Chord diagram of GO and KEGG analysis in Hypo-MDEGs. (C) Bubble chart of GO and KEGG enrichment analysis in Hyper-MDEGs. (D) Chord diagram of GO and KEGG analysis in Hyper-MDEGs. BP: biological process, CC: cell component, MF: molecular function.

In patients with TNBC, hypo-MDEGs were primarily involved in biological processes such as mitotic nuclear division, nuclear division, and organelle fission. These genes were predominantly associated with cellular components, including the spindle, centromeric chromosome regions, and kinetochore, while their molecular functions mainly involved microtubule binding, tubulin binding, and microtubule motor activity (Figure 5A). According to KEGG pathway analysis, hypo-MDEGs were enriched in progesterone-mediated oocyte maturation, cell cycle regulation, and oocyte meiosis (Figure 5A). The specific hypo-MDEGs associated with each GO term and KEGG pathway are listed in Table S6. Additionally, chord plots demonstrated 15 significantly enriched hypo-MDEGs (Figure 5B).

For hyper-MDEGs, GO enrichment analysis revealed that they were mainly involved in biological processes such as epithelial cell proliferation, response to peptide hormones, and sensory perception of pain. The cellular components associated with these genes included the collagen-containing extracellular matrix and basement membrane, while their molecular functions involved sulfur compound binding, heparin binding, and glycosaminoglycan binding (Figure 5C). However, KEGG pathway analysis did not identify significant enrichment for hyper-MDEGs. The hyper-MDEGs involved in each GO term are listed in Table S7. Additionally, 17 significantly enriched hyper-MDEGs were identified using a chord diagram (Figure 5D).

### 3.5 PPI network for identifying hub genes

**Figure 6:**
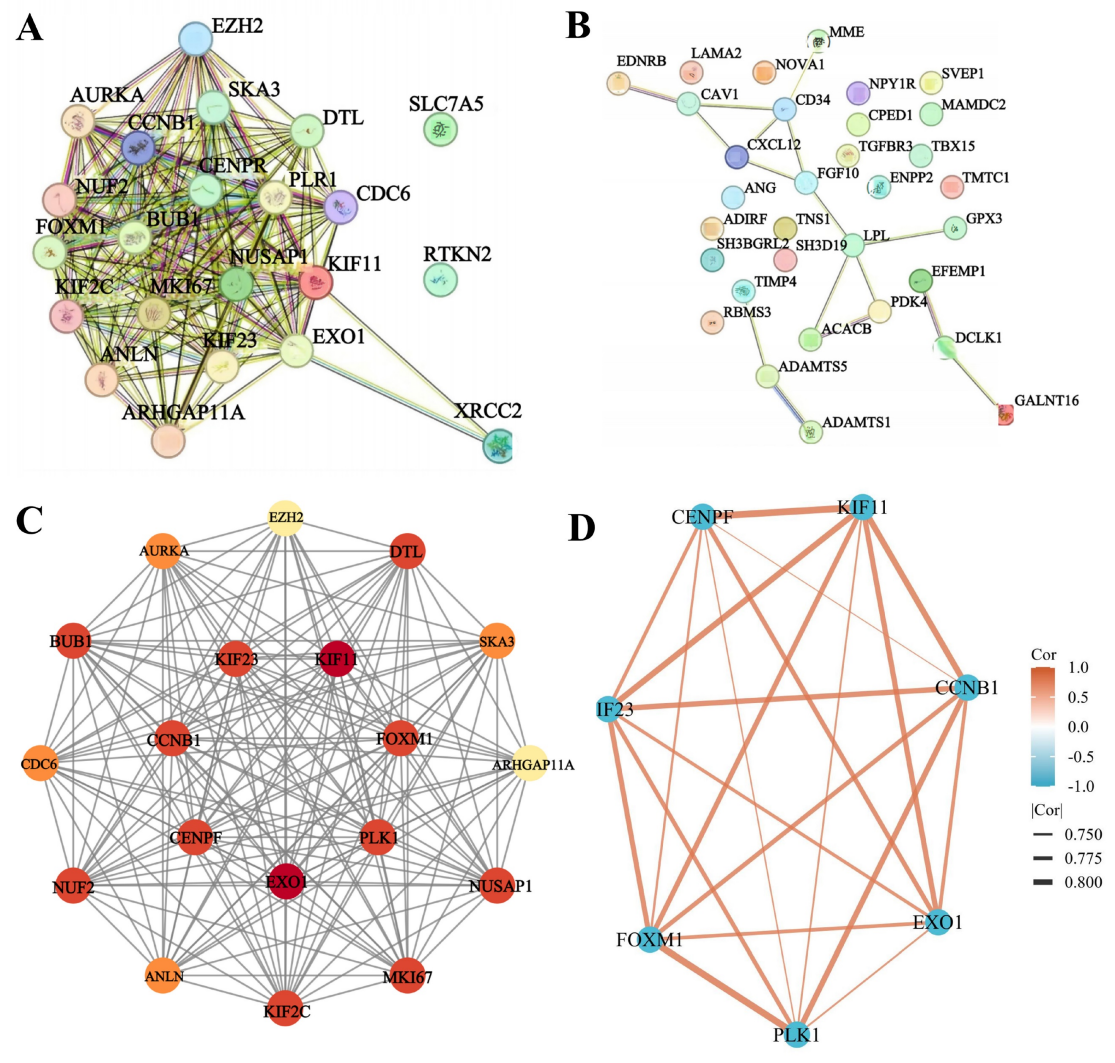
PPI network analysis for the identification of hub genes. (A) PPI network analysis of hypo-MDEGs from STRING database. (B) PPI network analysis of hyper-MDEGs from STRING database. (C) Circle map of significantly correlated genes of hypo-MDEGs highlighted by cytoscape software, the top 7 genes were located in the inner loop of the circle. (D) Network diagram of the correlation between 7 genes. The lines between the genes represent their relationships, with the thickness of the lines indicating the absolute value of the correlation coefficient, the thicker the line, the stronger the correlation. The color of the lines shows the direction of the correlation, with orange indicating a positive correlation and blue indicating a negative correlation.

The PPI network of hypo-MDEGs and hyper-MDEGs was predicted using the STRING database. The results showed that hypo-MDEGs exhibited complex protein interactions, forming 168 interaction pairs (Figure 6A). However, hyper-MDEGs demonstrated weak protein interactions, with only 16 interaction pairs (Figure 6B). Among the hypo-MDEGs, 19 genes exhibited strong interactions, and a network map of these genes was constructed using Cytoscape. The top 7 hub genes within the core network were identified using MCC, MNC, Degree, Closeness, and Radiality algorithms in CytoHubba, including *EXO1, KIF11*, *FOXM1, CENPF, CCNB1*, *PLK1*, and *KIF23* (Figure 6C). Furthermore, correlation network analysis of the expression levels of these 7 hub genes showed that the correlation coefficients were all greater than 0.7 (*p* < 0.05 for all genes), indicating a strong positive correlation between their expression levels (Figure 6D). These findings suggest that EXO1, KIF11, FOXM1, CENPF, CCNB1, PLK1, and KIF23 may serve as key hub genes in TNBC pathogenesis.

### 3.6 Effect of hub genes on diagnosis and prognostic of TNBC

**Figure 7:**
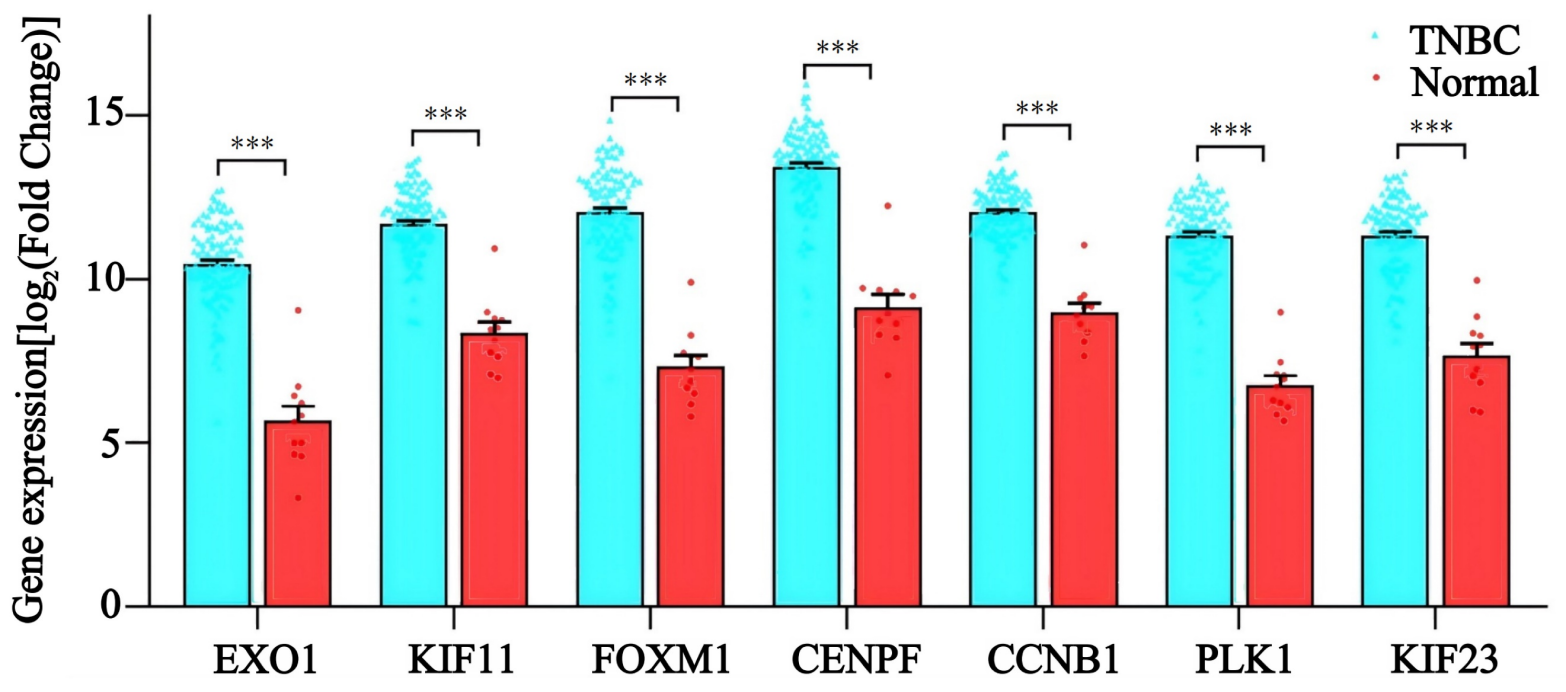
The mRNA levels of *EXO1*, *KIF11*, *FOXM1*, *CENPF, CCNB1, PLK1,* and *KIF23* in TNBC patients. ***: *p*< 0.001.

**Figure 8:**
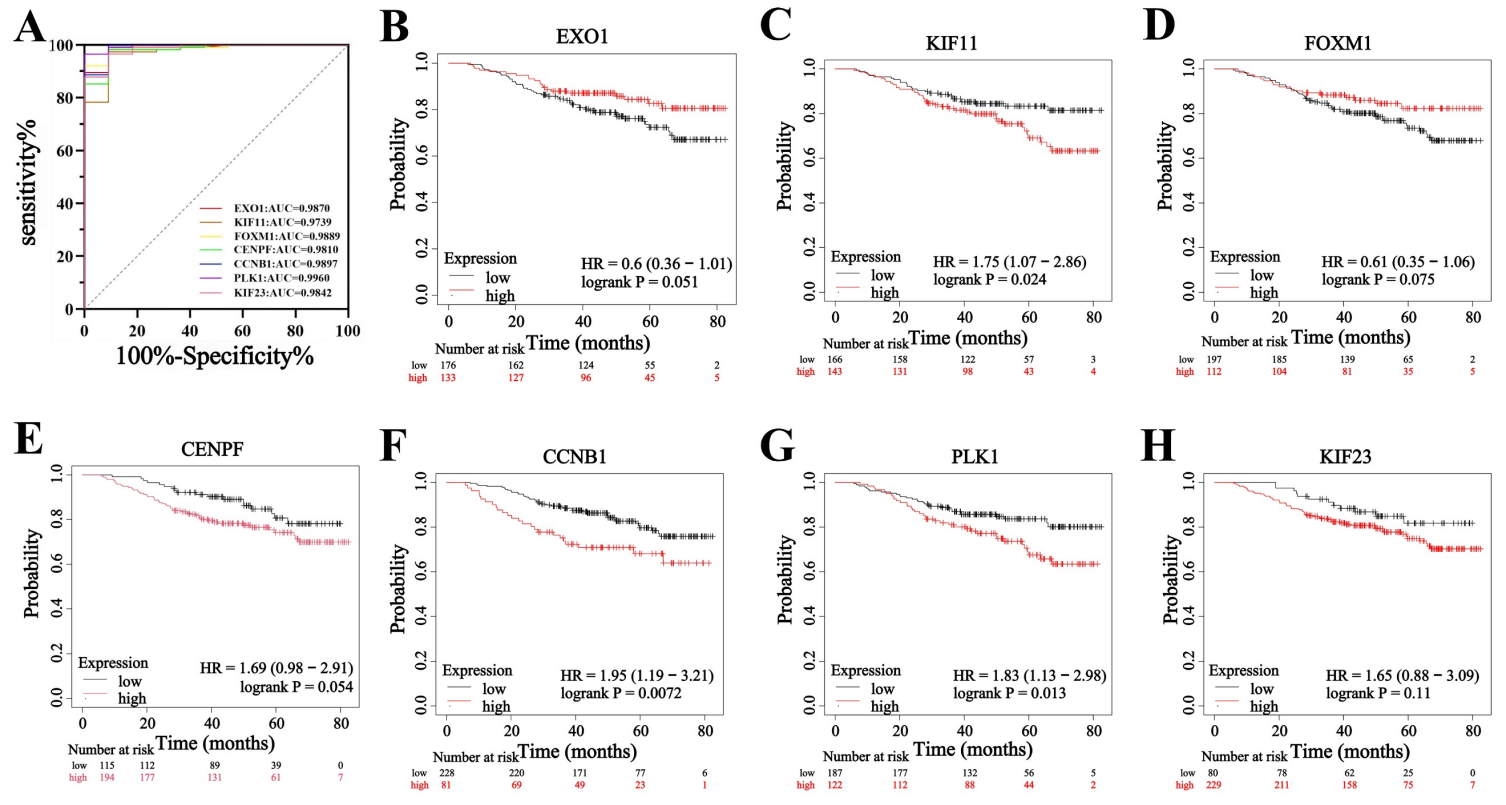
Effect of hub genes on diagnosis and prognostic of TNBC. (A) ROC analysis of *EXO1*, *KIF11*, *FOXM1*, *CENPF*, *CCNB1*, *PLK1* and *KIF23* expression in TNBC. (B-H) The KM curves of *EXO1*, *KIF11*, *FOXM1*, *CENPF*, *CCNB1*, *PLK1* and *KIF23* in TNBC.

To evaluate the diagnostic value and prognostic role of hub genes in TNBC, we first analyzed their expression levels in TCGA database. The results showed that the expression levels of *EXO1, KIF11*, *FOXM1, CENPF, CCNB1*, *PLK1*, and *KIF23* were significantly higher in TNBC tissues compared to adjacent noncancerous tissues (*p* < 0.05 for all genes) (Figure 7). These findings were consistent with the sequencing data from the present study. Next, ROC curve analysis was performed to assess the diagnostic potential of these hub genes. The results demonstrated that the AUC of the ROC curve for all seven hub genes exceeded 0.90 (*p* < 0.05 for all genes) (Figure 8A), suggesting that these genes hold strong diagnostic value for TNBC detection.

Furthermore, the prognostic significance of the seven hub genes in patients with TNBC was examined. The results revealed that *KIF11*, *CCNB1*, and *PLK1* had a hazard ratio (HR) greater than 1 when comparing high versus low expression groups (*p* < 0.05 for all genes) (Figure 8C, F, G), indicating that increased expression of these genes was associated with a poor prognosis in patients with TNBC. Additionally, the HR values for high versus low expression of *CENPF* and *KIF23* were greater than 1, but did not reach statistical significance (Figure 8E, H). Conversely, *EXO1* and *FOXM1* had HR values less than 1, yet these associations were also not statistically significant (*p* > 0.05) (Figure 8B, D). These findings suggest that while KIF11, CCNB1, and PLK1 may serve as key prognostic biomarkers for TNBC progression, *EXO1, FOXM1*,* CENPF*, and *KIF23* do not show a strong prognostic association in patients with TNBC.

### 3.7 Expression and methylation levels of hub genes in MDA-MB-231

**Figure 9:**
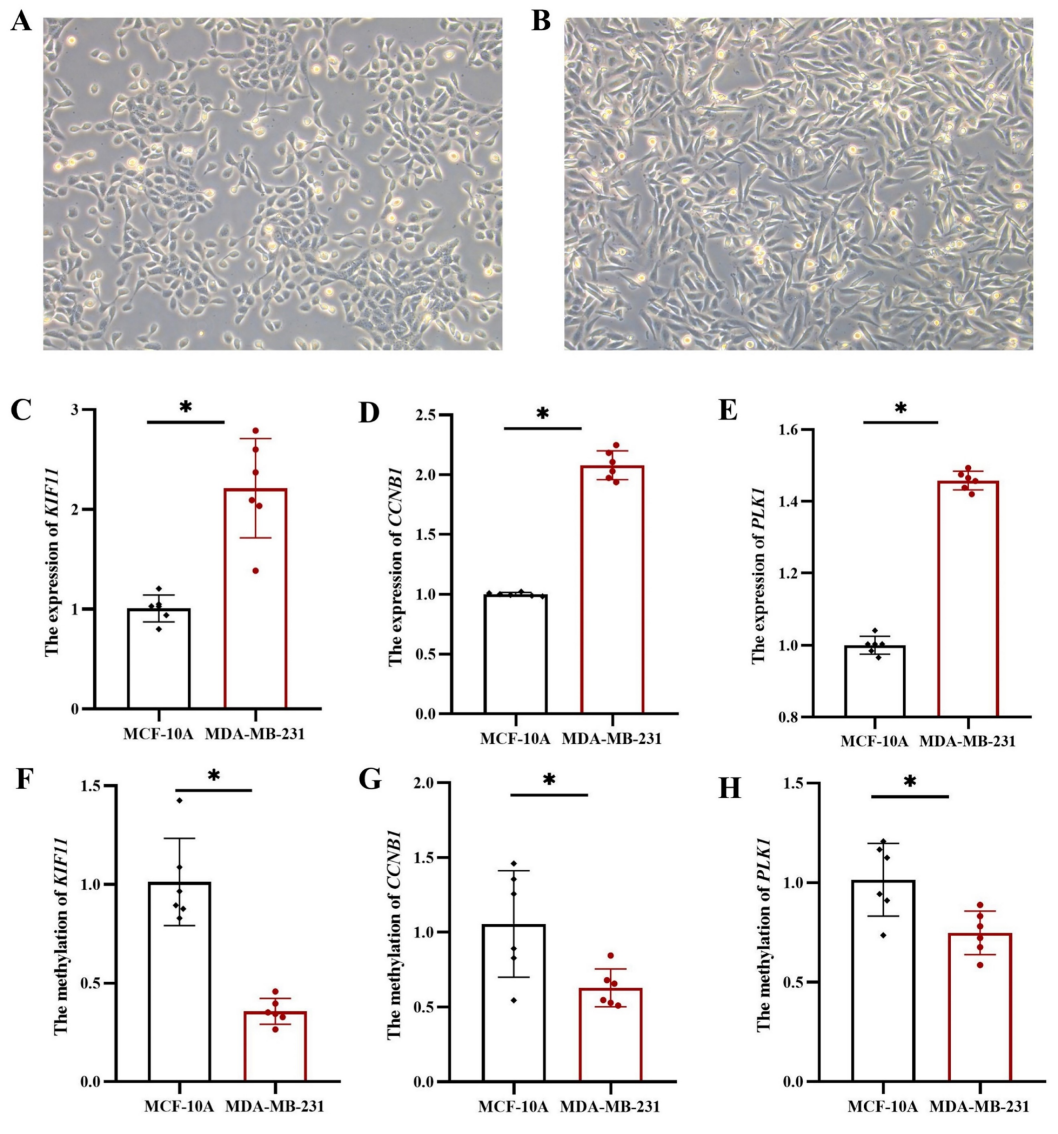
The mRNA and methylation levels of hub genes in MCF-10A and MDA-MB-231. (A) MCF-10A: normal breast epithelial cell line, (B) MDA-MB-231: TNBC cell line. The mRNA levels of *KIF11* (C), *CCNB1* (D) and *PLK1* (E). The methylation levels of *KIF11* (F), *CCNB1* (G) and *PLK1* (H). *: *p* < 0.05.

To further validate the expression and methylation levels of *KIF11*, *CCNB1*, and *PLK1*, we conducted experiments using TNBC cell line MDA-MB-231 and normal breast epithelial cell line MCF-10A. Morphological analysis showed moderate growth and good proliferation of MCF-10A (Figure 9A) and MDA-MB-231 (Figure 9B) cells in culture, fulfilling the experimental requirements. Gene expression analysis revealed that *KIF11*, *CCNB1*, and *PLK1* mRNA levels were significantly upregulated in MDA-MB-231 cells compared to MCF-10A cells (*p* < 0.05 for all genes) (Figure 9C-E). These results confirm that these three genes are highly expressed in TNBC cells, which is consistent with their elevated expression in TNBC patient samples, further supporting their role as prognostic markers for TNBC. Furthermore, DNA methylation levels in the promoter regions of *KIF11*, *CCNB1*, and *PLK1* were compared between MDA-MB-231 and MCF-10A cell lines. The results demonstrated significantly lower DNA methylation levels in the promoter regions of these three genes in MDA-MB-231 cells (*p* < 0.05 for all genes) (Figure 9F-H), suggesting that hypomethylation of *KIF11*, *CCNB1*, and *PLK1* contributes to their upregulated expression in TNBC.

## 4. Discussion

TNBC is a subtype of breast cancer characterized by the absence of ER, PR, and HER2 expression. TNBC exhibits high malignancy, early onset, rapid metastasis, poor prognosis, and an increased likelihood of recurrence. Due to the lack of effective targeted therapies, there is an urgent need to identify novel biomarkers that can improve TNBC prognosis and treatment strategies. DNA methylation has emerged as a critical area of epigenetic research in various malignancies, including breast cancer 19. However, therapeutic targets related to epigenetic regulation in TNBC remain limited in clinical practice. Advancements in this field have the potential to offer significant benefits for TNBC diagnosis and treatment. In this study, we analyzed RNA sequencing data and the GSE76250 dataset, identifying 98 upregulated genes and 87 downregulated genes that were differentially expressed in TNBC. Additionally, RRBS data analysis identified 5,059 hypermethylated genes and 3,234 hypomethylated genes. Further integration of DEGs and DMGs, followed by intersection and correlation analysis, identified 54 genes that exhibited a negative correlation between gene expression and DNA methylation status, including 22 hypo-MDEGs and 32 hyper-MDEGs.

To elucidate the biological functions of these 54 MDEGs, we performed GO and KEGG pathway enrichment analyses. The results indicated that hypo-MDEGs were primarily involved in spindle assembly, chromosome centromere organization, and centromere function. These genes played crucial roles in biological processes such as mitotic nuclear division and were associated with protein-binding functions, including microtubule binding, tubulin binding, and microtubule motor activity. Moreover, KEGG pathway analysis revealed that hypo-MDEGs were significantly enriched in the cell cycle pathway, progestin-mediated oocyte maturation pathway, and oocyte meiosis pathway. Spindles, centromeres, and microtubules are essential for mitotic progression, and abnormal mitosis can lead to dysregulated nuclear division, ultimately triggering tumorigenesis 20. Aberrant mitosis has been widely recognized as a hallmark of cancer progression 21. According to a study on TNBC mitosis, continuous BET protein activation promotes the sustained expression of cell cycle-related genes^22^, leading to mitotic catastrophe in cancer cells. The cell cycle pathway has been demonstrated to play a key role in TNBC progression 23, and its regulation is considered critical for effective cancer treatment 24 Additionally, hyper-MDEGs were predominantly involved in extracellular matrix and basement membrane composition, as well as binding to sulfur compounds and heparin. These genes played a significant role in epithelial cell proliferation and response to peptide hormones. Studies have shown that tumor epithelial cells can penetrate the basement membrane and interact with stromal fibroblasts, thereby enhancing breast cancer cell metastatic potential 25. We hypothesize that inhibiting the expression of these genes in TNBC cells may result in a weakened basement membrane, making tumor cells more susceptible to malignant expansion and metastasis.

To determine whether these genes interact, we used the STRING database and Cytoscape to identify 19 genes with strong interactions. We then applied the CytoHubba plugin in Cytoscape to identify the top 7 hub genes in TNBC, namely *EXO1, KIF11*, *FOXM1, CENPF, CCNB1*, *PLK1*, and *KIF23*, which exhibited strong positive expression correlations. The results indicate that the coordinated expression of these genes plays a vital role in mitosis and cell cycle regulation during tumor development. Previous studies have shown that FOXM1 activates the Wnt/β-catenin signaling pathway and enhances epithelial-mesenchymal transition (EMT) progression in TNBC by binding to the KIF23 transcriptional promoter 26. Another study demonstrated that FOXM1 promotes tumor progression and glycolysis in TNBC by regulating CENPA gene expression 27. However, limited research exists on interactions among these hub genes, underscoring the need for further investigations. Notably, these seven hub genes are classified as hypomethylated, highly expressed MDEGs (hypo-MDEGs), aligning with previous research 28 that reported an overall low level of DNA methylation in TNBC. We further assessed the diagnostic potential of these 7 hub genes and found that the AUC exceeded 0.9, indicating their significant diagnostic reference value for TNBC. Additionally, we evaluated the prognostic significance of these hub genes in patients with TNBC and observed that high expression of KIF11, CCNB1, and PLK1 was associated with poor prognosis. Our findings confirm that hypo-MDEG hub genes play a crucial role in both the diagnosis and prognosis of TNBC.

Previous studies have reported similar findings. KIF11 is a mitogenic kinesin 29 and a key regulator of the cell cycle. Knockdown of KIF11 leads to G2/M phase arrest, indicating its crucial role in TNBC tumor cell proliferation and self-renewal, both *in vitro* and *in vivo*. KIF11 is highly expressed in TNBC and is associated with shorter disease-free survival, making it a potential therapeutic target for drug-resistant TNBC 30. CCNB1 is significantly enriched in the cell cycle pathway and is highly expressed across multiple breast cancer subtypes, including luminal A, luminal B, HER2-positive, and TNBC. Its expression is strongly correlated with tumor pathological grade, disease stage, and metastasis 31. A study by Li *et al.* showed that CCNB1 is highly expressed in TNBC tissues and serves as a poor prognostic factor for patients with TNBC 32. PLK1 is a key regulator of cell division 33, and its inhibition has been shown to induce DNA damage, mitotic arrest, and ultimately cell death 34. The prognostic significance of PLK1 in breast cancer is subtype-dependent. While strong PLK1 expression is associated with longer survival in luminal breast cancer 35, its inhibition correlates with poor prognosis in TNBC 34. Consistent with these findings, our study demonstrated that patients with TNBC with high expression of *KIF11*, *CCNB1*, and *PLK1* had poor overall survival. These results suggest that aberrant DNA methylation may regulate gene expression and impact TNBC prognosis. Given that the elevated expression of *KIF11*, *CCNB1*, and *PLK1* is associated with an unfavorable TNBC prognosis, this study focused on analyzing their expression and methylation patterns. Our experimental results confirmed that *KIF11*, *CCNB1*, and *PLK1* were highly expressed with low methylation levels in TNBC cell lines. However, further biomolecular validation is required to confirm the protein-level expression of these genes and to further elucidate the relationship between gene expression and DNA methylation.

In addition, EXO1 is an exonuclease involved in cell cycle checkpoint regulation, replication fork maintenance, and post-replication DNA repair pathways 36. Previous studies have reported EXO1 expression in breast cancer to be associated with low methylation levels, and it has been found to be significantly enriched in the cell cycle pathway 37. CENPF, a member of the centromere protein family, plays a critical role in centromeric assembly and chromosome segregation. It is highly expressed in breast cancer and has been proposed as a diagnostic and prognostic marker 38. However, while the expression and methylation patterns of EXO1 and CENPF in TNBC remain unclear, our findings suggest that both EXO1 and CENPF exhibit high expression and low methylation status in TNBC. FOXM1 is a proliferative transcription factor that is widely expressed in actively dividing cells, including stem cells and tumor cells 39. Its expression is significantly upregulated in patients with TNBC compared with other breast cancer subtypes and normal breast tissues 40. However, studies have also shown that elevated FOXM1 expression has no significant effect on TNBC prognosis, whereas it plays a crucial prognostic role in ER+/HER2- breast cancer subtypes 41. This aligns with our findings in TNBC, where FOXM1 expression was not significantly associated with patient prognosis. KIF23, a key component of the central spindle complex, is essential for mitotic progression 42. It plays a crucial role in regulating cell division, DNA replication, and DNA damage repair 43. KIF23 is significantly upregulated in TNBC, where it activates the Wnt/β-catenin signaling pathway, thereby promoting EMT progression, migration, and metastasis, all of which are linked to poor TNBC prognosis 26. However, our study did not find statistically significant associations between the hub genes EXO1, CENPF, FOXM1, and KIF23 and TNBC prognosis.

In this study, we identified core genes significantly associated with TNBC diagnosis and prognosis through sequencing of clinical tissue samples, integration of multiple datasets, and comprehensive analysis of gene expression and methylation profiles. Our findings highlight the critical role of aberrant DNA methylation in regulating hub gene expression and prognosis, addressing limitations in previous genomic studies and providing potential targets for investigating the epigenetic molecular mechanisms underlying TNBC. However, this study has certain limitations that warrant further investigation. Future research will require analyses involving a larger cohort of clinical samples to strengthen these findings. Additionally, due to the heterogeneous nature of TNBC, further studies should explore the expression and methylation characteristics of core genes across specific TNBC subgroups to determine subtype-specific epigenetic alterations.

## 5. Conclusions

Collectively, our findings indicate that the 54 MDEGs are primarily enriched in mitosis-related processes, epithelial cell proliferation, and peptide hormone response functions, playing a central role in TNBC cell cycle pathways. Among these, *EXO1, KIF11*, *FOXM1, CENPF, CCNB1*, *PLK1*, and *KIF23* were identified as hub genes in TNBC, exhibiting strong positive correlations in their expression levels and demonstrating diagnostic significance for TNBC. Furthermore, we found that elevated expression of KIF11, CCNB1, and PLK1 was associated with poor TNBC prognosis, suggesting their potential as prognostic biomarkers. All seven hub genes identified in this study were found to be hypomethylated, reinforcing the significance of DNA methylation in TNBC pathogenesis. These findings contribute to an enhanced understanding of TNBC biomarkers, particularly regarding the epigenetic regulation of gene expression, and provide novel candidate biomarkers for the accurate diagnosis and targeted treatment of TNBC.

## Supplementary Material

Supplementary tables.
