# Supplementary material for: Identification of hub genes for the diagnosis and prognosis in triple negative breast cancer using transcriptome and differential methylation integration analysis
Source: J Cancer. 2025 Mar 3;16(6):2026–40. doi: 10.7150/jca.104472 (PMC11905416; doi:10.7150/jca.104472)
Supplement: Supplementary file 1 — Supplementary tables. [file jcav16p2026s1.zip › Table S1.docx]

Table S1: The sequence of primers used in RT-qPCR.

| Detection method | Genes | Sequence (5’-3’) |
| --- | --- | --- |
| RT-qPCR | *KIF11* | Fw: TGTAGATGCTGGTGTGGATTG |
|  |  | Rv: CTCTTTGTAACCAAGTGCTCTG |
|  | *CCNB1* | Fw: CTTTCGCCTGAGCCTATTTTG |
|  |  | Rv: TCCATCTTCTGCATCCACATC |
|  | *PLK1* | Fw: ACAGTTTCGAGGTGGATGTG |
|  |  | Rv: GGTTGATGTGCTTGGGAATAC |
|  | *ACTB* | Fw: ACCTTCTACAATGAGCTGCG |
|  |  | Rv: CCTGGATAGCAACGTACATGG |
| qMSP | *KIF11* | Fw: GTTAAGACGTCGAAAAGTTTTTTC |
|  |  | Rv: AAACGACTACACTAACCGAATAACG |
|  | *CCNB1* | Fw: GTTTTATTGTGGTTTCGTTTTTTTC |
|  |  | Rv: CTCCCTCCTTATTAACCTATTCGTA |
|  | *PLK1* | Fw: TTTCGGAGGTTTTAGTGGATTTAC |
|  |  | Rv: AAACATCTTCTCCCTCTAATACGAC |
|  | *ACTB* | Fw: AGAGTAAGAGAGGTATTTTTATTTTG |
|  |  | Rv: CAACAACACGAAATACTCCTC |

Abbreviations: RT‐qPCR, quantitative real‐time PCR; qMSP: quantitative methylation specific PCR. Fw: forward primers; Rv: reverse primers.
