# Supplementary material for: Identification of hub genes for the diagnosis and prognosis in triple negative breast cancer using transcriptome and differential methylation integration analysis
Source: J Cancer. 2025 Mar 3;16(6):2026–40. doi: 10.7150/jca.104472 (PMC11905416; doi:10.7150/jca.104472)
Supplement: Supplementary file 1 — Supplementary tables. [file jcav16p2026s1.zip › Table S4.docx]

Table S4: The map rate and bisulffte conversion rate of RRBS sequences

| Sample | RawReads | CleanReads | CleanReads Rate% | MappedReads | Mapping rate(%) | Conversion rate(%) |
| --- | --- | --- | --- | --- | --- | --- |
| CA_1 | 52291746 | 52290916 | 100 | 39114562 | 74.8 | 99.53 |
| CA_2 | 38612330 | 38612290 | 100 | 29852506 | 77.31 | 99.57 |
| CA_3 | 41596914 | 41596900 | 100 | 27106940 | 65.17 | 99.47 |
| CA_4 | 37795284 | 37791034 | 99.99 | 26422568 | 69.92 | 99.38 |
| CA_5 | 39455344 | 39455322 | 100 | 29673236 | 75.21 | 99.57 |
| CA_6 | 38602458 | 38602436 | 100 | 26483294 | 68.61 | 99.46 |
| NC_1 | 40722138 | 40722096 | 100 | 31521060 | 77.41 | 99.46 |
| NC_2 | 41312298 | 41311816 | 100 | 24609558 | 59.57 | 99.30 |
| NC_3 | 38742672 | 38742290 | 100 | 25881264 | 66.80 | 99.43 |
| NC_4 | 37956162 | 37956144 | 100 | 28319776 | 74.61 | 99.52 |
| NC_5 | 36930948 | 36930920 | 100 | 27527558 | 74.54 | 99.43 |
| NC_6 | 36759668 | 36759646 | 100 | 27207412 | 74.01 | 99.54 |
