# Supplementary material for: Identification of hub genes for the diagnosis and prognosis in triple negative breast cancer using transcriptome and differential methylation integration analysis
Source: J Cancer. 2025 Mar 3;16(6):2026–40. doi: 10.7150/jca.104472 (PMC11905416; doi:10.7150/jca.104472)
Supplement: Supplementary file 1 — Supplementary tables. [file jcav16p2026s1.zip › Table S6.docx]

Table S6: GO and KEGG enrichment analysis of hypo-MDEGs related with TNBC.

| **Category** | **Term** | **Description** | **P.adjust** | **Gene ID** | **Count** |
| --- | --- | --- | --- | --- | --- |
| BP | GO:0140014 | mitotic nuclear division | 6.23262E-16 | ANLN/PLK1/NUSAP1/MKI67/KIF2C/KIF11/KIF23/BUB1/CCNB1/CENPF/AURKA/CDC6/NUF2 | 13 |
| BP | GO:0000280 | nuclear division | 7.52082E-14 | ANLN/PLK1/NUSAP1/MKI67/KIF2C/KIF11/KIF23/BUB1/CCNB1/CENPF/AURKA/CDC6/NUF2 | 13 |
| BP | GO:0048285 | organelle fission | 1.8362E-13 | ANLN/PLK1/NUSAP1/MKI67/KIF2C/KIF11/KIF23/BUB1/CCNB1/CENPF/AURKA/CDC6/NUF2 | 13 |
| CC | GO:0005819 | spindle | 4.91058E-10 | PLK1/NUSAP1/KIF2C/KIF11/KIF23/CCNB1/SKA3/CENPF/AURKA/CDC6 | 10 |
| CC | GO:0000775 | chromosome,  centromeric region | 3.19058E-09 | PLK1/KIF2C/BUB1/CCNB1/SKA3/CENPF/NUF2/EZH2 | 8 |
| CC | GO:0000776 | kinetochore | 4.0287E-09 | PLK1/KIF2C/BUB1/CCNB1/SKA3/CENPF/NUF2 | 7 |
| MF | GO:0008017 | microtubule binding | 3.85725E-05 | PLK1/NUSAP1/KIF2C/KIF11/KIF23/CENPF | 6 |
| MF | GO:0015631 | tubulin binding | 0.00012699 | PLK1/NUSAP1/KIF2C/KIF11/KIF23/CENPF | 6 |
| MF | GO:0003777 | microtubule motor activity | 0.001695517 | KIF2C/KIF11/KIF23 | 3 |
| KEGG | hsa04914 | Progesterone-mediated  oocyte maturation | 8.93721E-05 | PLK1/BUB1/CCNB1/AURKA | 4 |
| KEGG | hsa04110 | Cell cycle | 8.93721E-05 | PLK1/BUB1/CCNB1/CDC6 | 4 |
| KEGG | hsa04114 | Oocyte meiosis | 8.93721E-05 | PLK1/BUB1/CCNB1/AURKA | 4 |
