# Supplementary material for: Identification of hub genes for the diagnosis and prognosis in triple negative breast cancer using transcriptome and differential methylation integration analysis
Source: J Cancer. 2025 Mar 3;16(6):2026–40. doi: 10.7150/jca.104472 (PMC11905416; doi:10.7150/jca.104472)
Supplement: Supplementary file 1 — Supplementary tables. [file jcav16p2026s1.zip › Table S7.docx]

Table S7: GO analysis of hyper-MDEGs related with TNBC.

| **Category** | **Term** | **Description** | **P.adjust** | **Gene ID** | **Count** |
| --- | --- | --- | --- | --- | --- |
| MF | GO:1901681 | sulfur compound binding | 1.43758E-06 | TGFBR3/CD34/ACACB/ANG/ADAMTS1/ADAMTS5/FGF10/LPL | 8 |
| MF | GO:0008201 | heparin binding | 2.22053E-05 | TGFBR3/ANG/ADAMTS1/ADAMTS5/FGF10/LPL | 6 |
| MF | GO:0005539 | glycosaminoglycan binding | 0.000102662 | TGFBR3/ANG/ADAMTS1/ADAMTS5/FGF10/LPL | 6 |
| CC | GO:0062023 | collagen-containing extracellular matrix | 0.000308141 | CXCL12/ANG/EFEMP1/ADAMTS1/ADAMTS5/FGF10/LAMA2 | 7 |
| BP | GO:0050673 | epithelial cell proliferation | 0.002610098 | TGFBR3/CD34/CXCL12/EDNRB/ANG/FGF10/CAV1 | 7 |
| BP | GO:0043434 | response to peptide hormone | 0.002610098 | TGFBR3/CXCL12/EDNRB/TIMP4/PDK4/CAV1/LPL | 7 |
| BP | GO:1901652 | response to peptide | 0.00341727 | TGFBR3/CXCL12/EDNRB/TIMP4/PDK4/CAV1/LPL | 7 |
| BP | GO:0019233 | sensory perception of pain | 0.005287951 | CXCL12/EDNRB/MME/NPY1R | 4 |
| CC | GO:0005604 | basement membrane | 0.015825206 | ANG/ADAMTS1/LAMA2 | 3 |
| MF | GO:0033218 | amide binding | 0.017772635 | ACACB/EDNRB/ANG/MME/NPY1R | 5 |
